# Effects of Nitrogen and Phosphorus Levels on Arbuscular Mycorrhizal Symbiosis and Associated Bacterial Communities in Culture

**DOI:** 10.3390/jof11110757

**Published:** 2025-10-22

**Authors:** Pengyuan Li, Jianbin Liu, Shubin Zhang, Yingbo Zhu, Xiaofang Yin, Lijun Xing, Dan Wei, Liang Jin

**Affiliations:** 1 College of Agronomy and Biological Science, Hebei Normal University of Science and Technology, Qinhuangdao 066004, China; m17332327984@163.com; 2 Institute of Plant Nutrition, Resources and Environment, Beijing Academy of Agriculture and Forestry Sciences, Beijing 100081, China; liujianbin1981@126.com (J.L.); xiaofang821010@sina.com (X.Y.); xing-lijun@163.com (L.X.); wd2087@163.com (D.W.); jinliang19762003@aliyun.com (L.J.)

**Keywords:** arbuscular mycorrhizal fungi, nitrogen, phosphorus, rhizosphere, microbial community

## Abstract

Arbuscular mycorrhizal (AM) fungi establish mutualistic symbioses with plant roots, enhancing plant growth and improving soil fertility through nutrient exchange. Among these, soil nitrogen (N) and phosphorus (P) are critical for symbiosis formation, directly influencing nutrient uptake and translocation within the symbiotic system. This study aimed to examine the regulatory roles of N and P levels on AM fungal development and associated bacterial communities in culture. Sorghum was used as the host plant in pot experiments with two AM fungi, *Rhizophagus irregularis* and *Funneliformis mosseae*, under varying N and P concentrations. The analyzed parameters included mycorrhizal colonization, propagule production, plant biomass, nutrient contents (N, P, and K), and bacterial community diversity. N3P1 treatment (150 mg/L N, 30 mg/L P) yielded the highest colonization rate, spore production, and arbuscule abundance in both AM fungal symbionts. At equivalent N and P concentrations, the N, P, and K contents in inoculated plants were significantly higher than those in controls. AM fungal inoculation markedly increased the bacterial diversity in the culture (Shannon index raised by 15.2–28.7%) and enriched beneficial taxa, such as *Bradyrhizobium* and *Pseudomonas*. N and P concentrations substantially influenced AM fungal symbiosis, with optimal development observed under N3P1 conditions. By regulating AM symbiotic establishment, N and P levels reshaped microbial community composition, providing theoretical guidance for industrialized AM fungal cultivation and inoculant production.

## 1. Introduction

Soil serves as a complex living system that supports the survival and activity of diverse microbial communities, including arbuscular mycorrhizal (AM) fungi [1]. AM fungi establish symbiotic associations with the majority of terrestrial plants [2], forming characteristic structures, such as intraradical hyphae, arbuscules, and vesicles within root cortical cells. Arbuscules act as the primary sites of bidirectional nutrient exchange, where host plants supply photosynthetic carbon compounds and AM fungi facilitate the uptake of mineral nutrients, particularly phosphorus and nitrogen [3]. This symbiotic relationship enhances plant nutrient acquisition and plays a vital role in the global carbon cycling [4].

As an essential macronutrient, phosphorus (P) contributes to cellular construction and regulates metabolic processes, profoundly influencing the establishment and function of AM symbiotic systems [5]. It is widely regarded as the dominant factor governing symbiosis. Both P fertilization and plant nutrient status can determine AM fungal colonization by regulating the host plant’s P-sensing system [6]. The P-sensing mechanism exhibits high sensitivity to soil P availability, and when available P exceeds the threshold above the optimal levels for plant growth, AM fungal colonization of roots is strongly suppressed. However, at optimal P levels AM symbiosis can simultaneously enhance plant productivity and improve P-use efficiency [7].

Nitrogen (N) is another critical growth-limiting nutrient [8] that has recently been studied for its role in AM fungal uptake and transport. AM fungi have substantial intrinsic N demand. Through their extensive hyphal networks, they can absorb various forms of N and deliver the assimilated N to host plants [9]. In N-limited environments, moderate N supplementation often promotes AM symbiosis [10]. However, N and P frequently exert complex or opposing effects on AM fungi. Low-to-moderate N levels generally stimulate AM fungal colonization, whereas P addition often inhibits colonization [11]. These contrasting effects may result from differences in host physiology, intrinsic nutrient demands, and soil nutrient transformation processes.

*Sorghum bicolor* L. is a highly mycorrhizal-responsive model plant and a globally important crop ranked fifth in total cultivation area among cereals [12]. It is widely used for food, feed, fiber, and biofuel production. Although primarily cultivated in low-phosphorus (P) soils and inherently efficient in nutrient use, sorghum remains heavily dependent on nitrogen (N) fertilization to achieve high grain yields in intensive agricultural systems [13]. Consequently, sorghum is an ideal model for studying the effects of nutrient status on plant growth and interactions with AM fungi species [14]. Furthermore, AM fungi colonization in sorghum roots has been demonstrated to enhance N and P uptake [15], and maintaining AM fungi diversity is confirmed to improve crop yield stability- a key attribute of sustainable agroecosystems [16].

The excessive use of chemical fertilizers has led to significant economic, environmental, and ecological losses, driving a global shift towards ecological intensification in agriculture [17]. As a core technology for green agricultural development, biofertilizers enhance nutrient cycling in the plant root zone, improve soil fertility, and promote mycorrhizal symbiosis. Through this symbiosis, AM fungi facilitate nutrient acquisition, particularly of less-mobile P, by solubilizing insoluble phosphates in the soil [18,19]. They also enhance the host plant’s uptake and transport of ammonium ions and improve the decomposition of organic matter.

Although the independent effects of N and P on AM fungi have been well-documented, their interactions remain poorly understood. For instance, in petunia-AM symbiosis, the inhibitory effect of P on colonization can be alleviated by N deprivation [20]. In tropical montane rainforests, both N and P additions can reduce AM fungal abundance, with combined N + P treatments causing the strongest decline in species abundance (39% reduction) [21]. High N addition significantly increased the abundance of most microbial genes in the soil and stimulated the microbial potential for P cycling in an alpine meadow soil [22]. Meanwhile, AM fungi can influence the composition of the rhizosphere microbial community. For instance, they recruit beneficial microorganisms in the alfalfa rhizosphere, thereby enhancing the complexity and stability of the microbial network [23]. Furthermore, the AM fungal symbiosis promotes the uptake of N and P, improving the soil nutrient acquisition efficiency of *Artemisia annua* [24].

Nevertheless, the mechanisms by which their interactions regulate AM development, propagule production, and functional efficiency remain poorly defined and require systematic study. This complexity is a key determinant of how AM fungi establish symbiosis and function under variable nutrient conditions [11,25]. Given the importance of AM fungi in plant nutrition, we hypothesized that optimal N/P concentration levels would promote better development of the AM fungal-sorghum symbiosis. Consequently, this study investigated the dynamic effects of different N and P levels on the development of two distinct AM fungal propagules, while also examining the response of the associated bacterial community. By elucidating the establishment and development of AM symbiosis under N-P regulation, this study provides essential theoretical and technical support for improving mycorrhizal applications and enhancing crop nutrient efficiency.

## 2. Materials and Methods

### 2.1. Test Materials

The host plant sorghum (*Sorghum bicolor* L. Moench) was obtained from Hebei Qingfengfeng Seed Co., Ltd., Shijiazhuang, China. The AM fungal inocula included *Funneliformis mosseae* (BGC XJ01) and *Rhizophagus irregularis* (BGC AH01), which were supplied by the Bank of Glomeromycota in China (BGC) at the Institute of Plant Nutrition, Resources, and Environment, Beijing Academy of Agriculture and Forestry Sciences. The growth substrate consisted of a 1:1 (*v*/*v*) mixture of perlite and river sand purchased from Beijing Huanmei Century Horticultural Supplies Co., Ltd, Beijing, China. The substrate was sterilized twice at 121 °C before experimental application. Cultivation was performed in plastic plug trays with an individual cell volume of 200 mL.

### 2.2. Experimental Design and Methods

The experiment was conducted in a greenhouse at the Institute of Plant Nutrition, Resources and Environment, Beijing Academy of Agriculture and Forestry Sciences, using a pot culture system. The temperature in the greenhouse during day and night is 28 °C and 23 °C, respectively, with a relative humidity of 60 ± 10% and natural light. Treatments included two AM fungal inoculations, *Funneliformis mosseae* (Fm) and *Rhizophagus irregularis* (Ri), and a non-mycorrhizal control (CK). For the inoculated treatments, 8 g of AM fungal inoculum was thoroughly mixed with 110 g of perlite–river sand substrate before pot filling. The ratio of inoculated bacteria dosage is 1:25 [26]. The control received 8 g of autoclaved inoculum (pre-cultured under identical conditions as the active treatments) together with 10 mL of inoculum filtrate. Seven factorial N-P combinations (Table 1) were applied within each AM fungal treatment (Fm and Ri) and CK, resulting in 21 treatments in total. Sampling was conducted at five time points: day 20, 40, 60, 80, and 100. To ensure three independent biological replicates for each treatment at every time point with no remaining samples afterward, each treatment consisted of 3 (replicates per time point) × 5 (time points) = 15 total replicates, resulting in a grand total of 315 experimental units. N and P supplementation was provided through an N-P-free Hoagland nutrient solution amended with sodium dihydrogen phosphate (NaH_2_PO_4_, P source) and ammonium nitrate ((NH_4_NO_3_, N source) at calibrated concentrations.

Eight sorghum seeds were sown per cell, and seedlings were thinned to five uniform individuals after germination. A weekly supply of 50 mL Hoagland nutrient solution containing the designated N/P levels was administered throughout cultivation. Sampling for indicator measurements was conducted on day 20, 40, 60, 80, and 100 after treatment. For each treatment, five seedlings per pot were sampled, with three replicates. At each harvest, shoots and roots were separated. Shoot samples were oven-dried at 75 °C to a constant weight for biomass determination. At 100 d, the above-ground samples were ground, and a 0.5 g portion was used to determine nitrogen, phosphorus, and potassium concentrations via atomic absorption spectroscopy. From the root system, 0.5 g of fresh material was taken for assessing AM fungal colonization, while the remaining root tissue was oven-dried to constant weight for biomass measurement. Concurrently, 5 g of fresh rhizosphere culture sample was stored at −80 °C for bacterial community analysis, and the remainder was air-dried for quantification of AM fungal propagules.

### 2.3. Indicators and Methods

#### 2.3.1. Determination of AM Fungal Colonization Rate and Propagule Density

Fixed sorghum roots were cut into ~1 cm segments and rinsed with distilled water. Root samples were stained using the vinegar-ink method [27], and 30 randomly selected root fragments were mounted on slides for microscopic observation. Colonization rates [14] were quantified under an optical microscope (400× magnification) using the gridline intersection method. According to established standards, infection intensity in root segments was classified as 0, <10%, <50%, >50%, or >90%, while the arbuscule abundance was classified as 0, <50%, or >50%. Data were entered into MYCOCALC software to calculate infection frequency (F), infection intensity (M), and arbuscule abundance (A). To calculate infection frequency (F), infection intensity (M), and arbuscule abundance (A).

Spores were extracted by wet sieving with 20 mL of air-dried culture [28]. The number of spores was recorded using a stereomicroscope (Leica Microsystems, Munich, Germany). For mycelium measurement, 5 g of air-dried culture was processed using vacuum filtration [29]. Mycelia were stained with 0.05% acid fuchsin, and density was determined microscopically.

#### 2.3.2. Determination of Biomass, Plant Nutrient Content, and Mycorrhizal Growth Response

Shoot and root biomass of sorghum were measured gravimetrically. The root-to-shoot ratio was calculated as root dry weight divided by shoot dry weight. The total nitrogen (TN), total phosphorus (TP), and total potassium (TK) contents in shoots were determined using the Kjeldahl method, molybdenum-antimony spectrophotometry, and flame photometry, respectively [30]. The mycorrhizal growth response was evaluated by comparing growth traits and physiological indices between inoculated and non-inoculated sorghum plants [14]. The MGR was calculated as follows: where SDW is the shoot dry weight. MGR (%) = [SDW (AM fungi-inoculated treatment) − mean SDW (Non-inoculated treatment)]/mean SDW (Non-inoculated treatment) × 100.

#### 2.3.3. Analysis of Bacterial Community Diversity in Culture Samples

High-throughput sequencing of culture samples was performed by Shanghai Majorbio Bio-Pharm Technology Co., Ltd. Genomic DNA was extracted using the FastDNA^®^ Spin Kit (MP Biomedicals, Santa Ana, CA, USA) for Soil and verified using 1% agarose gel electrophoresis. The bacterial 16S rRNA gene V3–V4 regions were amplified using primers 338F (5′-ACTCCTACGGGAGGCAGCA-3′) and 806R (5′-GGACTACHVGGGTWTCTAAT-3′). The PCR products were analyzed on 2% agarose gels, followed by library construction using the TruSeq^TM^ DNA Sample Prep Kit (Illumina, San Diego, CA, USA). Sequencing was performed on an Illumina MiSeq PE300 platform, and the data were analyzed for taxonomy, community diversity, and ecological associations.

#### 2.3.4. Statistical Data Analysis

Bacterial community diversity analysis was performed using the I-Sanger Cloud platform (Shanghai Majorbio Bio-Pharm Technology Co., Ltd, Shanghai, China). All experimental data were statistically analyzed using Microsoft Excel 2021, SPSS 26.0, and DPS software (v9.50) for ANOVA. Statistical analyses were performed separately at each sampling time point for all measured variables, including mycorrhizal colonization rate, propagule density, plant biomass, and nutrient content. All datasets were first subjected to tests for normality (Shapiro–Wilk test) and homogeneity of variances (Levene’s test). For data meeting the assumptions of normality and homoscedasticity, a one-way analysis of variance (One-way ANOVA) was used to examine the effects of different N and P levels under the same AM fungal inoculation condition. A two-way ANOVA was applied to assess the overall effects of N/P levels, AM fungal inoculation species, and their interactions. When ANOVA indicated significant differences (*p* < 0.05), post hoc multiple comparisons were conducted using Tukey’s Honest Significant Difference (HSD) test to identify specific differences among treatment groups. For data that did not meet the assumptions for parametric tests, the non-parametric Kruskal–Wallis H test was employed, followed by Duncan’s new multiple range test for pairwise comparisons. Graphical representations were generated using Origin 2021.

## 3. Results

### 3.1. Dynamic Effects of N and P Levels on AM Fungal Colonization and Propagule Development

#### 3.1.1. Dynamic Effects of N and P Levels on Colonization Parameters (M% and A%)

**Figure 1:**
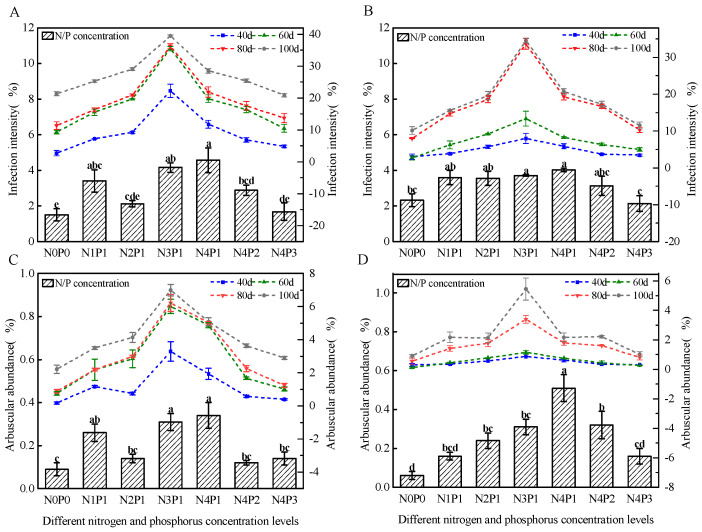
Spatio-temporal variations in AM fungal infection intensity and arbuscular abundance under different N and P levels. (**A**,**B**) Infection intensity of the Fm strain (**A**) and the Ri strain (**B**) across sampling time points. The left Y-axis (bar graph) represents the value at 20 d, and the right Y-axis (line graph) displays the values at 40 d, 60 d, 80 d, and 100 d. (**C**,**D**) Arbuscular abundance of the Fm strain (**C**) and the Ri strain (**D**) across sampling time points. The left Y-axis (bar graph) indicates the value at 20 d, while the right Y-axis (line graph) shows the values at the subsequent time points (40 d, 60 d, 80 d, and 100 d). Different letters indicate significant differences according to Tukey’s HSD test (*p* < 0.05).

Both inoculated AM fungi (Fm and Ri) successfully colonized sorghum roots, forming functional mycorrhizal symbioses, whereas no colonization was observed in the non-inoculated control. Across different N and P concentrations, infection density (M%) and arbuscule abundance (A%) increased progressively with time, reaching maximum levels at 100 days (Figure 1). For Fm, M% and A% increased steadily from 20 to 60 d and increased more slowly from 60 to 100 d. For Ri, the increases were gradual from 20 to 60 d but accelerated between 60 and 100 d. At N3P1 (150 mg/L N, 30 mg/L P), M% and A% of both fungi were consistently higher than those under other treatments.

#### 3.1.2. Effects of N and P Levels on AM Fungal Propagule Development (Spore Density and Hyphal Density)

**Figure 2:**
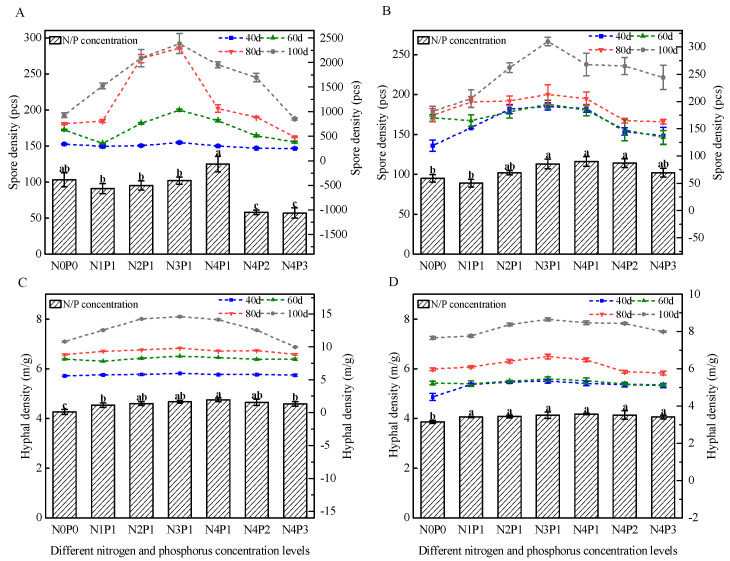
Spatio-temporal variations in AM fungal spore density and mycelial density under different N and P levels. (**A**,**B**) Spore density of the Fm strain (**A**) and the Ri strain (**B**) at different time points. The left Y-axis (bar graph) indicates the infection intensity at 20 d, while the right Y-axis (line graph) shows the infection intensity at 40 d, 60 d, 80 d, and 100 d. (**C**,**D**) Mycelial density of the Fm strain (**C**) and the Ri strain (**D**) at different time points. The left Y-axis (bar graph) indicates the arbuscular abundance at 20 d, whereas the right Y-axis (line graph) represents the arbuscular abundance at 40 d, 60 d, 80 d, and 100 d. Different letters indicate significant differences according to Tukey’s HSD test (*p* < 0.05).

At 40, 60, 80, and 100 d, the spore density under N3P1 (150 mg/L N, 30 mg/L P) was consistently greater than that in other treatments. At a given P level, a moderate N increase favored spore formation, whereas excessive N suppressed it. The number of spores increased with cultivation time, peaking at 100 d. Hyphal density demonstrated trends similar to spore density. Notably, under N3P1 conditions, both spore production and hyphal density of Fm and Ri were significantly higher than those in the other treatments (Figure 2).

### 3.2. Effects of Different N and P Levels and AM Fungi Inoculation on Sorghum Biomass, Root-to-Shoot Ratio and Nutrient Content

#### 3.2.1. Effects of Different N and P Levels and AM Fungi Inoculation on Sorghum Biomass and Root-to-Shoot Ratio

Under all N and P treatments, inoculated groups (Ri and Fm) showed significantly higher total biomass and root-to-shoot ratios than the non-inoculated control (CK) at 40, 60, 80, and 100 d. The results indicated that the N3P1 treatment (150 mg/L N and 30 mg/L P) most effectively promoted host growth and achieved the highest mycorrhizal effect (Table 2).

#### 3.2.2. Effects of N and P Concentrations and AM Fungi Inoculation on Plant N, P, and K Content

**Figure 3:**
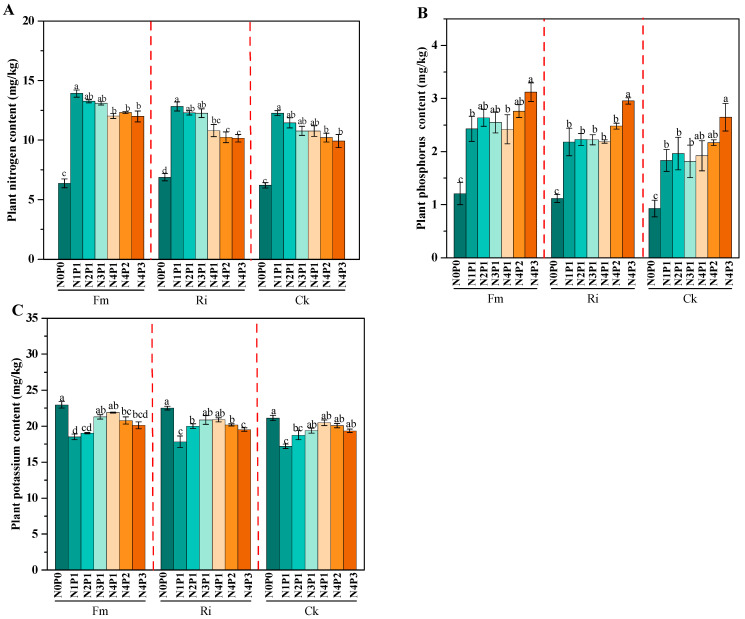
Plant N, P, and K content under different N and P levels and AM fungal inoculation treatments. Panels **A**, **B**, and **C** represent plant nitrogen content, phosphorus content, and potassium content, respectively, under different fungal inoculation and N/P level treatments. Different letters indicate significant differences according to Tukey’s HSD test (*p* < 0.05).

At identical N and P concentrations, the inoculated sorghum plants accumulated significantly higher N, P, and K contents than the non-inoculated controls, with statistically significant differences (*p* < 0.05). The Fm-inoculated plants reached a maximum nitrogen content of 13.91 mg/kg under the high-N, low-P treatment (N1P1), whereas the P content peaked at 3.12 mg/kg under N4P3. The K content reached its highest value of 22.98 mg/kg under nutrient-poor conditions (N0P0). At constant N, elevated P significantly increased the plant P concentration but suppressed N uptake. Under constant P, the reduced N generally lowered the plant N concentration without notable effects on P or K. The K content displayed a “low-nutrient advantage”, with all fungal strains achieving the peak levels (21.14–22.98 mg/kg) under N0P0. In contrast, a high N supply (N1P1) significantly reduced the K content (minimum of 17.21 mg/kg) (Figure 3).

### 3.3. Effects of N and P Levels on Bacterial Communities in AM Fungal Cultures

#### 3.3.1. Bacterial Microbial Diversity of Different AM Fungal Cultures at Different N and P Levels

**Figure 4:**
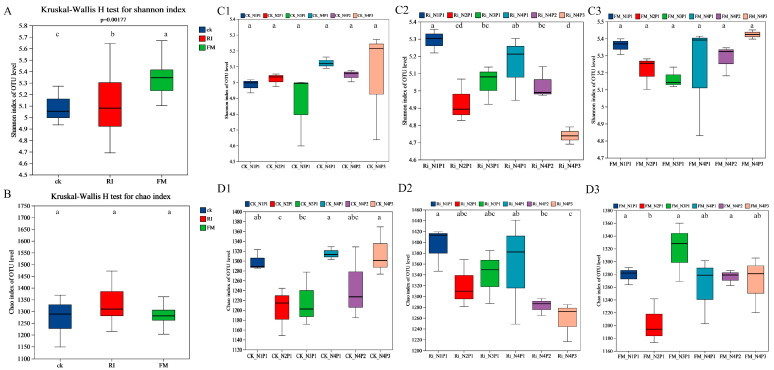
Bacterial α-diversity indices under different treatments. (**A**,**B**) Intergroup comparisons of Shannon and Chao1 indices of bacterial communities under different inoculation treatments. (**C**,**D**) Intergroup comparisons of Shannon and Chao1 indices under different inoculation and concentration treatments. The panels (**C1**–**C3**) and (**D1**–**D3**) represent the results at different sampling time points (e.g., 40, 60, and 80 days), respectively. Intergroup differences were analyzed using the Kruskal-Wallis rank-sum test (*p* < 0.05).

**Figure 5:**
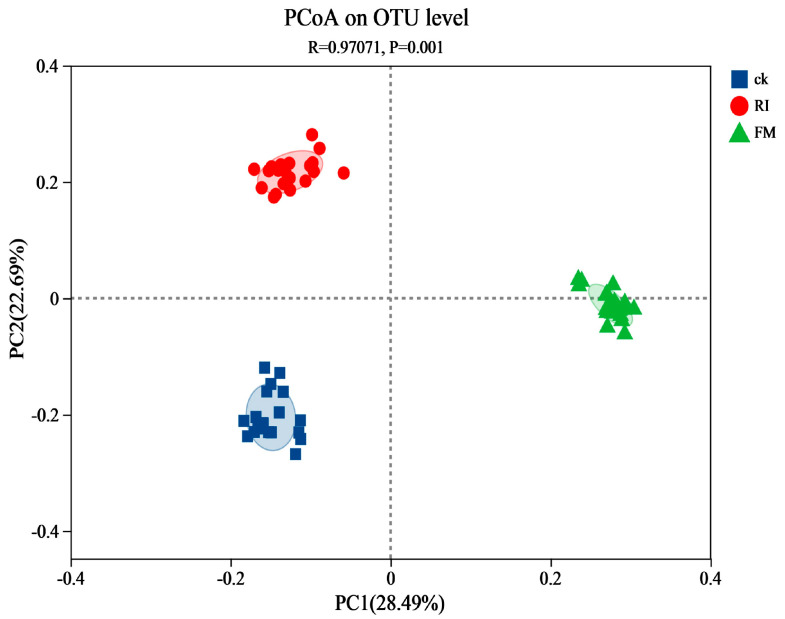
Principal coordinate analysis of bacterial community composition in AM fungal cultures at different N and P concentrations. The shaded regions (red, blue, green) delineate the clusters of bacterial communities associated with three distinct treatment groups defined by specific N and P concentration combinations.

In total, 40 phyla, 144 classes, 283 orders, 471 families, and 893 genera were detected across treatments. Comparative analysis of diversity indices revealed significant inter-group differences in the Shannon index (*p* = 0.0017; *p* < 0.05), whereas Chao1 showed no significant variation (*p* > 0.05). The Fm-inoculated cultures exhibited the highest bacterial richness under N3P1, whereas Ri-inoculated cultures reached the maximum diversity and richness under N1P1 (Figure 4). Principal coordinate analysis (PERMANOVA, R2 = 0.97071, *p* = 0.0010) demonstrated distinct bacterial community structures among Fm, Ri, and CK treatments. These findings indicated that AM fungal inoculation exerted a significant impact on bacterial community assembly in cultures (Figure 5).

#### 3.3.2. Effects of N Concentrations on Bacterial Community Diversity in AM Fungal Cultures

**Figure 6:**
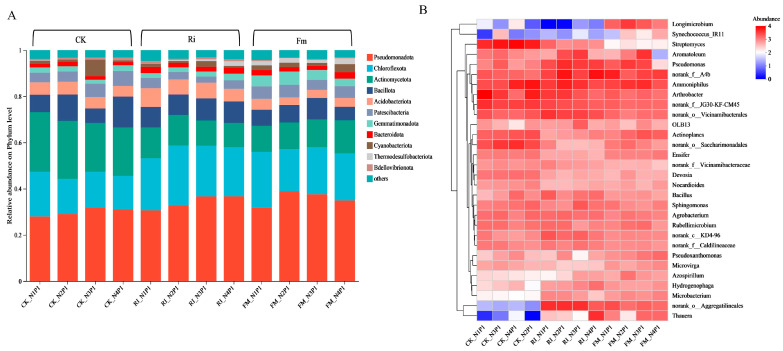
Bacterial community composition of AM fungal cultures at different N concentrations. (**A**) Relative abundances of bacterial communities at the phylum level. (**B**) Relative abundances of bacterial communities at the genus level across different N concentration gradients.

At the phylum level (Figure 6A), the relative abundance of *Pseudomonadota* in Ri-inoculated cultures decreased progressively with increasing N concentrations (36.72%, 36.67%, 32.95%, and 30.62%). In contrast, *Actinomycetota* abundance increased with higher N levels in both Ri-inoculated and CK treatments. Fm-inoculated treatments exhibited a significant decline in *Actinomycetota*, decreasing sequentially to 14.33%, 11.98%, 11.57%, and 11.33% under elevated N concentrations. The dominant phylum *Chloroflexota* reached its maximum relative abundance (25.74%) in Ri-inoculated cultures under N2P1 conditions.

Genus-level heatmap analysis (Figure 6B) showed that inoculation with Fm and Ri significantly enhanced the relative abundance of *Thauera* and *norank_o__Aggregatilineales* compared with CK across N gradients. *Longimicrobium* and *Synechococcus_IR11* were more abundant in Fm-inoculated treatments than in CK and Ri. Under high N (N1P1), *Arthrobacter* dominated in CK, whereas Bacillus predominated in Ri. At moderate N (N2P1–N3P1), Ri significantly increased the abundance of *Pseudomonas*. At N3P1, Fm treatments presented peak abundances of *Actinoplanes* and *Ammoniphilus*. Ri-inoculated cultures under low N (N4P1) showed the highest *Thauera* abundance.

#### 3.3.3. Effects of P Concentrations on Bacterial Community Diversity in AM Fungal Cultures

**Figure 7:**
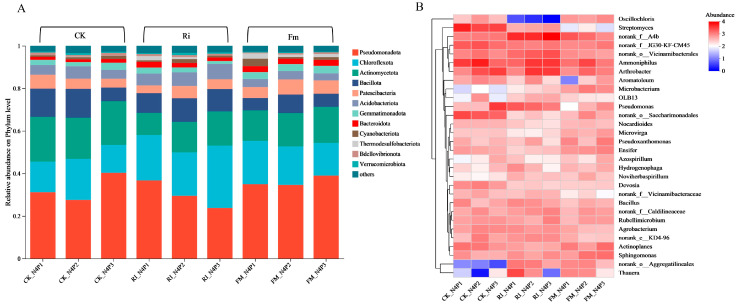
Bacterial community composition of AM fungal cultures at different P concentrations. (**A**) Relative abundances of bacterial communities at the phylum level. (**B**) Relative abundances of bacterial communities at the genus level across different P concentration gradients.

At the phylum level (Figure 7A), *Pseudomonadota* (40.30%), *Chloroflexota* (29.31%), and *Actinomycetota* (21.06%) were dominant across treatments. In Ri-inoculated cultures, *Pseudomonadota* decreased with higher P concentrations (36.72%, 29.49%, and 23.75%), whereas *Actinomycetota* increased (10.46%, 14.30%, and 16.08%). Under N4P3, Ri exhibited the highest abundance of *Chloroflexota*. Concurrently, Fm inoculation resulted in a progressive increase in *Actinomycetota* (14.33%, 15.67%, and 16.93%) with elevated P content.

Genus-level analysis (Figure 7B) revealed that both Fm and Ri inoculation significantly increased *Thauera* and *norank_o__Aggregatilineales* under varying P levels compared with CK. *Oscillochloris* was more abundant in Ri than in CK and Fm. In Fm cultures, *Arthrobacter* and *Microbacterium* were enriched, peaking under N4P2. Sphingomonas increased with increasing P content, whereas Thauera decreased. In Ri cultures, the unclassified *norank_f__A4b* and *Arthrobacter* peaked at N4P3. Elevated P also increased *Ammoniphilus* and *Microbacterium* but reduced *Bacillus* and *Pseudomonas*. Under low P, both Fm and Ri maintained higher *Thauera* abundance than CK. These results confirmed that different AM fungal strains selectively enriched bacterial taxa with distinct metabolic strategies by altering resource availability under varying N/P conditions.

### 3.4. Correlation Analysis of AM Fungi, Plant, and Bacterial Community Diversity

**Figure 8:**
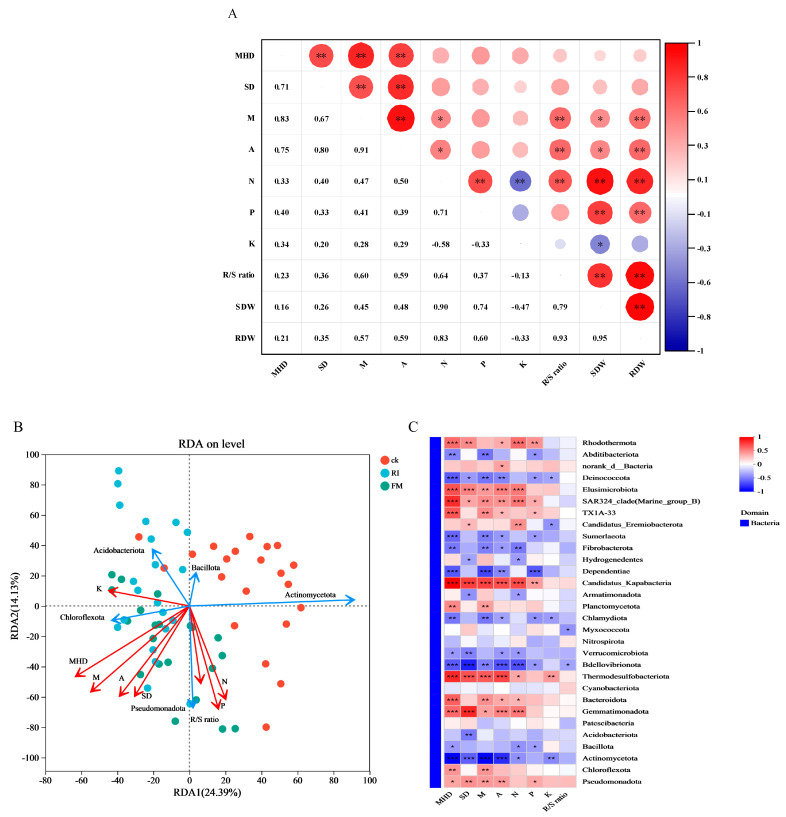
Correlation analysis among AM fungi, plants, and bacterial communities in culture. (**A**) Correlations among AM fungal propagules, root-to-shoot ratio, and plant N, P, and K contents under different N and P concentrations. (**B**) Redundancy analysis of bacterial communities at the phylum level. (**C**) Heatmap showing correlations between bacterial communities at the phylum level. Significant differences across treatments are indicated (* *p* < 0.05; ** *p* < 0.01; *** *p* < 0.001). Abbreviations: MHD, hyphal density; SD, spore density; M, colonization intensity; A, arbuscular abundance; N/P/K, plant nitrogen/phosphorus/potassium content; R/S ratio, root-to-shoot ratio; SDW, shoot dry weight; RDW, root dry weight.

Correlation analysis (Figure 8A) indicated that hyphal density and spore density in AM fungal cultures were positively correlated with both arbuscule abundance and root colonization intensity across different N/P treatments. Arbuscule abundance and colonization intensity were positively correlated with plant nitrogen content, indicating that enhanced mycorrhizal colonization improved nutrient acquisition efficiency in host plants. Plant P content was positively correlated with both shoot and root dry weights, confirming that P is the key limiting factor for biomass accumulation. In contrast, K content was significantly negatively correlated with nitrogen levels and shoot biomass, reflecting a dilution effect during rapid shoot growth. Redundancy analysis (RDA) and correlation heatmap visualization (Figure 8B,C) revealed significant negative correlations between mycorrhizal parameters (colonization intensity, arbuscule abundance, hyphal density, spore density, root-to-shoot ratio) and plant N/P/K contents with *Actinomycetota* and *Bacillota*. Conversely, colonization intensity, arbuscule abundance, hyphal density, spore density, and root-to-shoot ratio were positively correlated with *Chloroflexota* and *Pseudomonadota*. Hyphal density and plant K content were significantly negatively correlated with *Acidobacteriota*.

## 4. Discussion

### 4.1. Effects of Different N and P Levels on AM Fungal Propagule Development

#### 4.1.1. Effects of Different Nitrogen and Phosphorus Levels on AM Fungal Colonization Rate

The growth and nutrient translocation of AM fungi require substantial energy, which can be sustained by enhancing host nutrient uptake capacity. This relationship establishes a dynamic carbon-nutrient trade-off equilibrium [25]. More importantly, it reveals that the synergistic regulation of N and P nutrition is key to optimizing this exchange efficiency. In this study, the N3P1 treatment (N: 150 mg/L; P: 30 mg/L) significantly increased sporulation and hyphal growth compared with other nutrient treatments (*p* < 0.05), indicating N-mediated positive feedback on mycorrhizal symbiosis. When P availability was fixed at 30 mg/L, the optimal N input (150 mg/L) promoted synchronized development between host plants and AM fungi. Enhanced N availability stimulated N uptake by AM fungi, driving plants to allocate more C to mycorrhizae. Conventional wisdom holds that high N or P levels suppress mycorrhizal symbiosis [31]. Our findings, however, challenge this paradigm by demonstrating that the key lies in the balanced supply of nutrients rather than the absolute quantity of any single element. This is consistent with recent studies showing that N or P addition can alter the intensity of the priming effect by modulating the alignment between soil C:N:P ratios and microbial stoichiometric demands [32]. The optimal N3P1 treatment (N:P ratio = 5:1) in our study fell within an ideal stoichiometric range, which likely simultaneously activated the plant’s demand for both N and P. Consequently, this may have prevented the plant from reducing carbon allocation to AM fungi by perceiving them as a “carbon cost” under conditions of single-nutrient surplus. This finding aligns with the recently proposed “conditional symbiosis” theory [33,34]. Therefore, our results not only support classical theories but also emphasize the critical importance of shifting from a single-nutrient perspective to an N-P coupling framework when evaluating mycorrhizal responses.

Numerous studies have confirmed a strong correlation between P availability and mycorrhizal colonization, with variations in P concentration significantly influencing root infection dynamics and regulating hyphal development and sporulation [35]. When soil P concentrations fall below plant demand, host dependence on AM fungi can exhibit a significant decrease. Under these conditions, the C cost of maintaining mycorrhizal symbiosis outweighs its nutritional benefits, leading to reduced allocation of photosynthates to AM fungi and consequent declines in colonization intensity and hyphal density [36]. This reflects the plant’s optimal carbon allocation strategy. Consequently, inhibitory effects on root colonization were observed. Moreover, the variation in the biomass of the two AM fungal species (*Funneliformis mosseae* and *Rhizophagus intraradices*) under different phosphorus levels showed strong consistency with their respective root colonization capacities in host plants.

Recent studies have suggested that the underlying mechanism of high-P inhibition may not only stem from plant carbon allocation adjustments but also be associated with alterations in root exudate composition or the downregulation of fungal P-sensing signaling pathways [37]. Our experimental results further support this perspective, implying that future research should move beyond the simple correlation of “sufficient P leads to reduced symbiosis” and delve into the phosphorus signaling mechanisms within plant-fungal interactions.

#### 4.1.2. Synergistic Relationships Between AM Fungal Propagation/Development and Plant Biomass as Well as N, P, and K

Studies have reported [38] that when soil P concentration falls below a critical threshold (e.g., <30 mg/L), host plant roots can develop a “phosphorus depletion zone”. Under such P-deficient conditions, AM fungi overcome P diffusion limitations through extensive extraradical hyphal networks, thereby significantly enhancing P acquisition efficiency [39]. In this study, mycorrhizal colonization intensity, arbuscular abundance, and propagule production under the 30 mg/L P + 150 mg/L N treatment were significantly higher than those in the control. Concurrently, host sorghum plants exhibited significantly greater biomass and root-to-shoot ratios, indicating increased C allocation to sustain mycorrhizal development and enhanced N and P uptake, thereby establishing mutualistic symbiotic relationships.

Studies have demonstrated that host plants can reduce their dependence on AM fungi when soil phosphorus availability meets nutritional requirements [40]. High phosphorus conditions suppress the expression of arbuscule-specific phosphate transporter genes, thereby impairing P transport by AM fungi [41]. Concurrently, C allocation to AM fungi is reduced, resulting in hyphal degeneration and decreased sporulation [42]. This is because N enrichment enhances photosynthetic C assimilation, providing additional resources to sustain fungal hyphal function and promoting beneficial symbiotic efficiency [43]. Mutualistic symbiosis between AM fungi and plants significantly improves plant nutritional status and promotes growth and yield. However, the performance of AM fungi is closely dependent on soil nutrient availability [44]. Under high-P conditions (>60 mg/L), the decline in AM fungal colonization, indicated by reduced hyphal density and spore production, could not be reversed even with sufficient nitrogen supply, demonstrating P-mediated suppression of symbiosis.

Currently, many commercial AM fungal inoculants often utilize P-rich media during multiplication to guarantee host plant growth. However, this practice may unintentionally select for strains adapted to high-P environments, potentially compromising their symbiotic competitiveness in practical field conditions with low to medium P availability [45]. The results of this study demonstrate that the balanced nutrient formulation represented by the N3P1 concentration level is an ideal parameter for efficiently producing high-quality inoculants. Under this formulation, AM fungi (including both the Fm and Ri strains) simultaneously achieved high spore density, mycelial density, and root colonization rates, thereby ensuring high viability and colonization potential of the inoculant. Therefore, we recommend that “balanced N and P supply under moderate phosphorus levels” be adopted as one of the key standardized parameters in the industrial fermentation or pot-based multiplication of AM fungi, ultimately enhancing the overall quality of inoculant products from the source.

#### 4.1.3. Impact of Cultured Bacterial Communities on AM Fungi

Plant roots serve as the primary interface for plant–microbe interactions. AM fungal inoculation not only optimizes root system architecture but also recruits diverse microbial communities, thereby enhancing soil nutrient bioavailability [46]. This outcome suggests that under nutrient stress (particularly P deficiency), plants preferentially allocated photosynthetic C to AM fungal species capable of alleviating stress. Such selective C allocation leads to differences in soil nutrient sensitivity among AM fungi, thereby shaping the structure of diverse microbial communities [38]. This process may act as an upstream driver of shifts in the rhizospheric bacterial communities.

The results further demonstrated that inoculation with AM fungi, particularly Fm and Ri strains, significantly enhanced the relative abundances of several dominant bacterial phyla in the culture system. This indicated that AM fungal inoculation under varying N and P concentrations enriched bacterial groups with distinct ecological functions [47]. For example, *Pseudomonadota* can play a critical role in modulating bacterial community structure and functions, such as nitrogen cycling, owing to its exceptional metabolic versatility. Similarly, *Actinomycetota* can contribute to soil biogeochemical cycling through efficient decomposition of organic matter and transformation into plant-available nutrients [48]. The present findings corroborate the existing evidence that AM fungi can substantially alter rhizospheric microbial composition. Representative cases include restructuring alfalfa rhizobacterial communities under phosphorus-deficient conditions [49] and selectively enriching beneficial microbes in maize rhizospheres in lanthanum-contaminated soils [50]. Under chromium stress, AM fungi enhance microbial interaction networks by recruiting dominant rhizospheric phyla such as *Pseudomonadota*, Actinobacteria, and Chloroflexi. Moreover, AM fungi recruit bacteria, including Pseudomonas and Bacillus, which secrete phytase (appA), thereby degrading phytate phosphorus into plant-available forms [51].

Low-to-medium fertility soils represent the optimal scenario for AM fungi to exert their community regulatory functions. More importantly, this study confirms that AM fungi can enhance the microbial network by modulating dominant phyla. The inconsistent effectiveness of traditional AMF inoculants is partly attributable to their neglect of the integrity of the rhizosphere microbiome. We propose that future inoculant development should focus on creating composite microbial agents containing a “core AM fungus strain + key growth-promoting bacteria.” This shift from single-strain to consortia-based inoculants aims to mimic the efficient rhizosphere micro-ecosystem found in nature, thereby achieving potent synergistic effects.

## 5. Conclusions

This study employed sorghum as the host plant to investigate the effects of seven N/P concentration gradients on the development of arbuscular mycorrhizal (AM) fungi symbiosis, sorghum growth, and rhizobacterial communities through pot experiments. The results demonstrated that the N3P1 treatment (150 mg/L N, 30 mg/L P) provided the optimal conditions for the symbiotic development of both AM fungal strains. Under this condition, AM fungi maximally promoted sorghum growth and nutrient acquisition, while actively modulating the rhizosphere microbiota. These findings offer a theoretical foundation for the industrial-scale cultivation of AM fungi and the production of microbial inoculants, along with critical theoretical and technical support for sustainable sorghum cultivation. However, the precise economic thresholds of AM fungal symbiosis under N/P interaction remain incompletely elucidated and require further in-depth investigation.

## Figures and Tables

**Table 1:** Experimental treatments with different nitrogen and phosphorus levels.

Treatment	N (mg/L)	P (mg/L)
N0P0	0	0
N1P1	210	30
N2P1	180	30
N3P1	150	30
N4P1	120	30
N4P2	120	60
N4P3	120	90

**Table 2:** Sorghum biomass, root-to-shoot ratio, and mycorrhizal growth response under differential N/P regimes with AM fungal inoculation.

Inoculation Treatment	N/P Concentration	Total Dry Weight (g)	Root Shoot Ratio	Mycorrhizal Growth Response
Fm	N0P0	1.08 ± 0.06 d	0.12 ± 0.03 c	0.05
	N1P1	4.34 ± 0.31 ab	0.15 ± 0.02 b	0.11
	N2P1	4.32 ± 0.23 ab	0.15 ± 0.01 b	0.11
	N3P1	4.46 ± 0.2 a	0.19 ± 0 a	0.12
	N4P1	3.89 ± 0.17 c	0.15 ± 0.01 b	0.09
	N4P2	3.93 ± 0.41 bc	0.14 ± 0.01 bc	0.08
	N4P3	3.96 ± 0.17 bc	0.14 ± 0.01 bc	0.07
Ri	N0P0	1.04 ± 0.1 c	0.12 ± 0.02 c	0.02
	N1P1	4.36 ± 0.32 a	0.14 ± 0.01 bc	0.11
	N2P1	4.36 ± 0.2 a	0.15 ± 0.02 b	0.11
	N3P1	4.48 ± 0.26 c	0.19 ± 0.02 a	0.12
	N4P1	3.69 ± 0.21 b	0.15 ± 0 b	0.04
	N4P2	3.86 ± 0.23 b	0.14 ± 0.02 bc	0.06
	N4P3	3.85 ± 0.47 b	0.14 ± 0.02 bc	0.06
Ck	N0P0	1.02 ± 0.07 b	0.11 ± 0 c	-
	N1P1	3.88 ± 0.25 a	0.14 ± 0.02 b	-
	N2P1	3.87 ± 0.31 a	0.15 ± 0.01 b	-
	N3P1	3.95 ± 0.75 a	0.18 ± 0 a	-
	N4P1	3.55 ± 0.27 a	0.14 ± 0.02 bc	-
	N4P2	3.62 ± 0.59 a	0.13 ± 0.03 bc	-
	N4P3	3.66 ± 0.38 a	0.13 ± 0.02 bc	-

Note: Data are presented as mean ± SE (*n* = 3). Different lowercase letters within the same column indicate statistically significant differences (*p* < 0.05) among treatments under the same inoculation condition.

## Data Availability

The original contributions presented in this study are included in the article. The raw high-throughput sequencing data have been deposited in the NCBI database under BioProject accession number PRJNA1310815.
